# Reasons for and Outcomes of Medicare Part D Appeals for Denied GLP‐1 Receptor Agonist Claims

**DOI:** 10.1111/dom.71201

**Published:** 2026-08-10

**Authors:** Matthew J. Klebanoff, John C. Lin, Judith A. Long, Eric Bressman, Jalpa A. Doshi

**Affiliations:** ^1^ University of Pennsylvania Perelman School of Medicine Philadelphia Pennsylvania USA; ^2^ Leonard Davis Institute of Health Economics Philadelphia Pennsylvania USA; ^3^ Philadelphia VA Center for Healthcare Evaluation, Research, and Promotion Philadelphia Pennsylvania USA

**Keywords:** antidiabetic drug, GLP‐1 analogue, health economics, pharmaco‐economics, type 2 diabetes

## 1 Introduction

Glucagon‐like peptide‐1 receptor agonists (GLP‐1s) have transformed the management of diabetes and related metabolic conditions. In Medicare Part D, claims for GLP‐1s have more than tripled in recent years, from 6.1 million in 2020 to over 21 million claims in 2024 [1]. Expanding use has been accompanied by rising spending and prescribing beyond covered indications [2, 3, 4]. In response, Medicare Part D plans have introduced more restrictive coverage policies [5].

If coverage restrictions intensify and access to GLP‐1s becomes constrained, the Part D appeals process offers beneficiaries and clinicians a mechanism to contest denials. When plans deny coverage for a drug, the decision can be appealed and, if unsuccessful, escalated to an independent entity for reconsideration (i.e., level 2 appeal) [6]. Level 2 appeals may therefore become an increasingly important recourse for clinicians seeking to prescribe GLP‐1s for their patients. However, it remains unknown whether this process meaningfully facilitates access to GLP‐1s. We analysed Medicare Part D level 2 appeals for GLP‐1s from 2020 to 2025 to characterise the types of coverage determinations that lead to level 2 appeals, the outcomes of these appeals and the reasons for denied appeals.

## 2 Methods

We analysed publicly available level 2 appeals in Medicare Part D using the Centers for Medicare and Medicaid Services (CMS) Appeals Decision Search. This national dataset of level 2 appeals contains data for the entire US [7]. For each level 2 appeal, the dataset includes a date, appeal outcome, condition(s) for which the drug was prescribed, drug name, rationale for the appeal decision and coverage rules that informed the decision. We studied semaglutide and tirzepatide products indicated for diabetes, including injectable semaglutide (Ozempic, approved December 2017), oral semaglutide (Rybelsus, approved September 2019) and injectable tirzepatide (Mounjaro, approved May 2022) (eTable 1). We excluded their brand name counterparts that are approved for weight loss, as Part D is statutorily prohibited from covering weight loss drugs and these drugs only recently gained indications beyond weight loss.

We developed an automated approach to classifying the purpose of the initial coverage determination that led to each appeal (e.g., requests for formulary exceptions) (eMethods 1), using string‐based text searches (eTable 2). When this approach flagged more than one purpose, the following hierarchy was applied to determine the primary purpose: (1) tiering exception, (2) formulary exception, (3) step therapy exception, (4) quantity limit exception, (5) payment‐related issue and (6) request for prior authorization. For unfavourable appeals, we also extracted the rationale for denial (eMethods 2, eTable 3). When more than one reason was flagged, the following hierarchy was applied to determine the primary reason: (1) Part D exclusion for weight loss, (2) off‐label use, (3) utilisation management criteria not met and (4) administrative issue. To validate the automated approach to classification, two investigators independently classified 150 appeal summaries and compared these classifications to automated results (eMethods 3). For a subsample of appeals denied due to off‐label prescribing, we manually classified the indications for which GLP‐1 s were prescribed.

From quarter 1 of 2020 to quarter 2 of 2025, we calculated percentages of appeals originating from different types of initial coverage determinations, percentages with favourable outcomes and, among unsuccessful appeals, percentages denied for different reasons. The Cochran‐Armitage test for trends was used to investigate trends in the purpose of initial coverage determinations and the primary reason for denials over time (measured in quarters).

## 3 Results

We identified 8821 appeals from 2020 (quarter 1) to 2025 (quarter 2): 4764 for injectable semaglutide, 528 for oral semaglutide and 3529 for tirzepatide.

Over the study period, appeals increasingly originated from claims involving requests for prior authorization (Figure 1). For injectable semaglutide, prior authorization accounted for only 10% of appeals in 2020 versus 90% in 2025. Oral semaglutide followed a similar trajectory, with 73% of appeals involving requests for prior authorization by 2025. For tirzepatide, prior authorization already accounted for 51% of appeals in 2022 (its first year on the market) and led to 81% of appeals by 2025.

**FIGURE 1:**
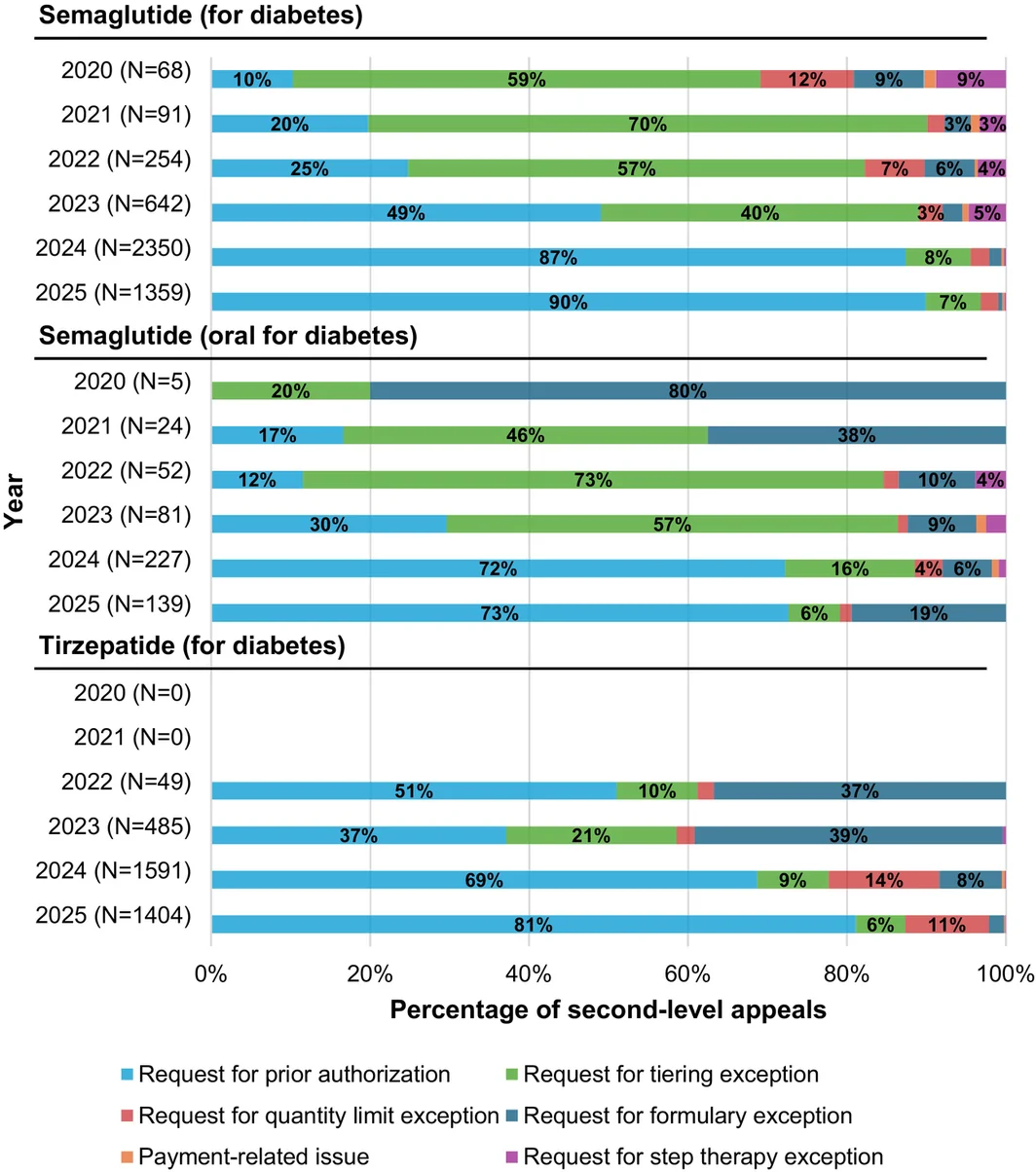
Types of initial coverage determinations resulting in level 2 appeals for semaglutide and tirzepatide products approved for type 2 diabetes, quarter 1 of 2020 to quarter 2 of 2025. This figure shows the percentage of level 2 appeals that resulted from different types of initial coverage determinations, across each drug. There were no tirzepatide appeals prior to 2022 because this drug gained FDA approval in May 2022. This figure includes only appeals from quarter 1 of 2020 to quarter 2 of 2025, due to availability of appeals data when this analysis was conducted. eMethods contain descriptions of each type of initial coverage determination. Due to space constraints, this figure does not include labels for any percentages ≤ 2%.

Appeals rarely succeeded (Figure 2). In 2025, fewer than 1 in 5 appeals had favourable outcomes (15% for injectable semaglutide, 19% for oral semaglutide and 11% for tirzepatide). The leading reason for denial shifted over time. In 2020, appeals were frequently denied for not meeting utilisation management criteria (78% for injectable semaglutide and 100% for oral semaglutide). By 2025, however, the most common reason for denial was off‐label use (53%, 40% and 48%, respectively). Each of the aforementioned temporal trends—for types of coverage determinations and reasons for denials—was statistically significant, with *p* < 0.001.

**FIGURE 2:**
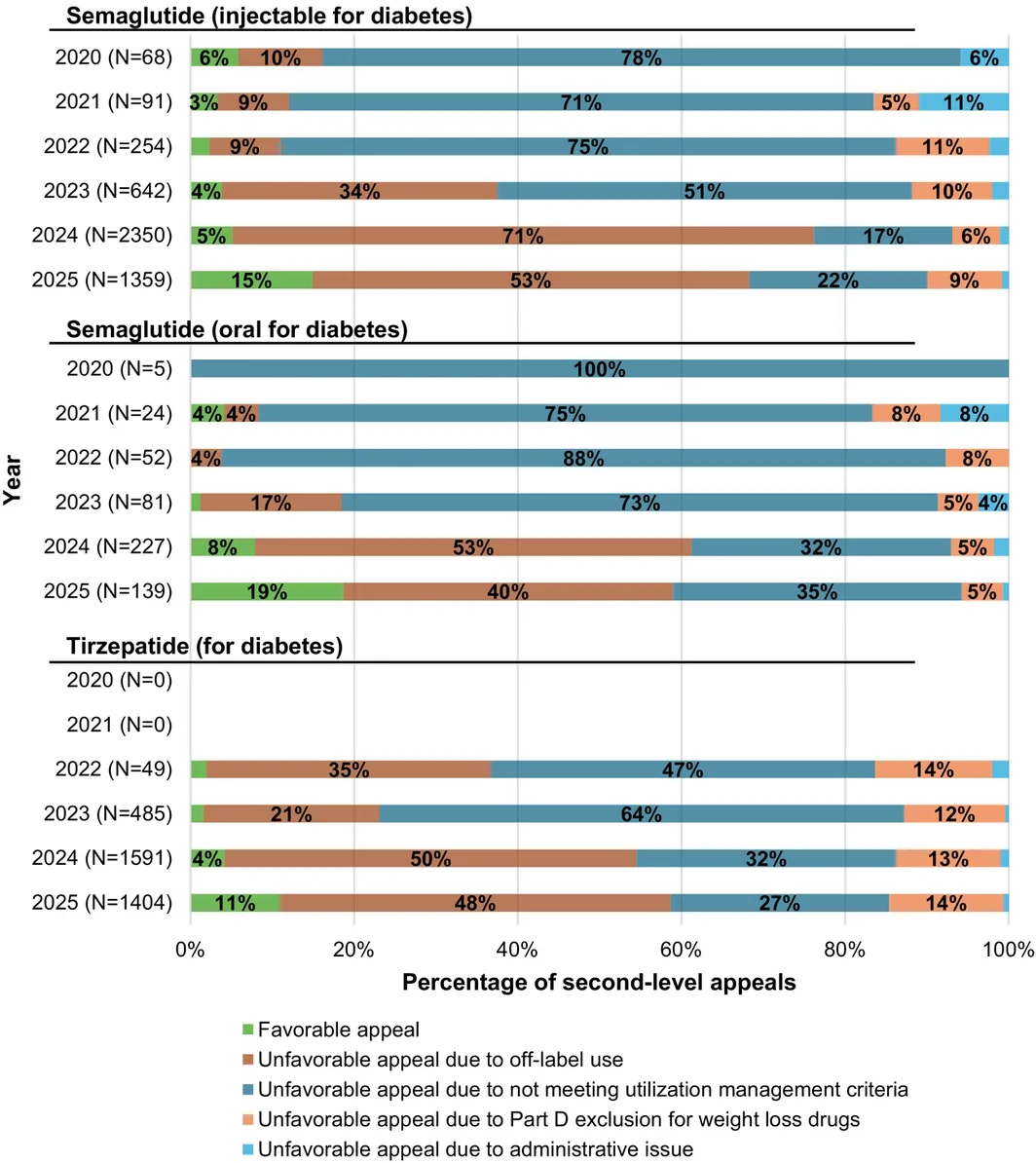
Outcomes of level 2 appeals for semaglutide and tirzepatide products approved for type 2 diabetes, quarter 1 of 2020 to quarter 2 of 2025. This figure shows the percentage of level 2 appeals in each year with favourable versus unfavourable outcomes, across each drug. For unfavourable appeals, reasons for denial are also shown. eMethods describe the approach to classifying reasons for unfavourable appeals. This figure includes only appeals from quarter 1 of 2020 to quarter 2 of 2025, due to availability of appeals data when this analysis was conducted. Due to space constraints, this figure does not include labels for any percentages (≤ 2%).

For appeals where the rationale for denial was off‐label use, approximately one‐quarter listed obesity or overweight as the sole indication (25% for injectable semaglutide, 28% for oral semaglutide and 22% for tirzepatide) (eTable 4). Other common indications for which GLP‐1 s were prescribed off‐label included prediabetes and cardiovascular disease.

Automated classification showed high agreement with manual review for both the initial coverage determination type (98.0% observed agreement; Cohen's *κ* = 0.96) and reason for appeal denial (92.0%; Cohen's *κ* = 0.87).

## 4 Discussion

From 2020 to 2025, appeals increasingly resulted from prior authorization; success rates for appeals remained persistently low; and off‐label prescribing became the leading reason for denial. The infrequent success of level 2 appeals suggests that reversal is uncommon once cases reach the independent review stage.

The increase in appeals resulting from prior authorization coincides with the widespread introduction of prior authorization requirements for GLP‐1 s among Part D plans, which took place in 2024 and 2025 [5]. In addition, the rise in off‐label prescribing as the leading reason for denial—accounting for approximately half of unfavourable appeals across all GLP‐1 s by 2025—may reflect growing demand for GLP‐1 s beyond currently covered indications in Medicare. Starting July 1, 2026, Medicare launched the GLP‐1 Bridge Program, which may reduce denials due to off‐label use by providing GLP‐1 coverage to beneficiaries with a body mass index (BMI) of at least 35 kg/m^2^, as well as those meeting lower BMI thresholds who have certain comorbid conditions [8].

This study has limitations. Level 2 appeals represent a fraction of Part D claim denials because most are resolved at the plan level or never appealed [6]. We could not assess appeal rates relative to overall prescribing or denial rates and appeal summaries had limited clinical information, precluding evaluation of the appropriateness of decisions. The CMS appeals dataset also lacked information on age, sex, socioeconomic status, geographic location and plan type (i.e., standalone versus Medicare Advantage Part D plan). Lastly, we could not account for the potential influence of obesity stigma on denials.

Nevertheless, this study offers a rare window into GLP‐1 coverage barriers for the Medicare population that are otherwise difficult to observe. As eligibility for GLP‐1s expands in Medicare, monitoring of coverage decisions and patient access will be essential.

## Funding

Dr. Klebanoff reported funding from K12DK133995 (NIDDK) outside of the submitted work.

## Conflicts of Interest

The authors declare no conflicts of interest.

## Supporting information


**Data S1:** dom71201‐sup‐0001‐Supinfo.docx. **eTable 1**. Semaglutide and tirzepatide products approved for the treatment of diabetes.
**eMethods 1**. Approach to classifying types of coverage determinations.
**eTable 2**. Search terms used to classify type of coverage determination.
**eMethods 2**. Approach to classifying reasons for unfavourable outcomes of appeals.
**eTable 3**. Search terms used to classify reason for unfavourable appeal outcome.
**eMethods 3**. Validation of approach to classifying types of initial coverage determinations and reasons for unfavourable outcomes.
**eTable 4**. Indications for GLP‐1 prescriptions among a subsample of appeals denied due to off‐label prescribing in 2024 and 2025.

## Data Availability

The data that support the findings of this study are available in the CMS Appeals Decision Search at https://www.cms.gov/medicare/appeals‐grievances/appeals‐decision‐search‐part‐c‐d.
