# Supplementary material for: Reasons for and Outcomes of Medicare Part D Appeals for Denied GLP‐1 Receptor Agonist Claims
Source: Diabetes Obes Metab. 2026 Aug 10;28(11):10878–81. doi: 10.1111/dom.71201 (PMC13646333; doi:10.1111/dom.71201)
Supplement: Supplementary file 1 [file DOM-28-10878-s001.docx]

**eTable 1. Semaglutide and tirzepatide products approved for the treatment of diabetes**

| **Drug name** | **Brand name** | **Route of administration** | **Manufacturer** | **Date of initial FDA approval** | **Indication(s)** |
| --- | --- | --- | --- | --- | --- |
| Semaglutide | Ozempic^®^ | Subcutaneous | Novo Nordisk Inc | 12/5/2017 | Type 2 diabetes mellitus |
| Semaglutide | Rybelsus^®^ | Oral | Novo Nordisk Inc | 9/20/2019 | Type 2 diabetes mellitus |
| Tirzepatide | Mounjaro^®^ | Subcutaneous | Eli Lilly and Co | 5/13/2022 | Type 2 diabetes mellitus |

This table includes the semaglutide and tirzepatide products included in the analysis. The analysis included semaglutide and tirzepatide products that are currently available in the U.S. and approved for treatment of diabetes. Abbreviations: FDA, Food & Drug Administration

**eMethods 1. Approach to classifying types of coverage determinations**

We manually reviewed a subset of level 2 appeals for GLP-1s to determine the major types of coverage determinations that led to the appeals.

Most coverage determinations fit into the following categories:

- Requests for prior authorization;
- Requests for formulary exceptions, which involved requests for a drug not on the plan’s formulary;
- Requests for exceptions to step therapy, which involved requests for drug coverage without first trialing a therapeutic alternative;
- Requests for exceptions to quantity limits, which involved requests for dispense of a higher quantity than permitted by plan rules;
- Requests for exceptions to tiering, which involved requests to obtain a non-preferred drug in a higher cost-sharing tier at more favorable cost-sharing terms applicable to drugs in lower cost-sharing tiers;
- And requests for reimbursement, which typically involved disputes over the amount of cost-sharing required for a prescription.

Based on review of a subset of appeal summaries, we identified phrases that tended to appear in certain types of appeals. This process began with using a large language model (ChatGPT, GPT-4o) to identify a list of candidate search terms, based on review of a subset of 200 appeal summaries. The research team then built upon this list and iteratively developed a comprehensive list of phrases to use as search terms. The final list of search terms was validated by two team members (eMethods 3).

When >1 coverage determination type indicator was positive, the following hierarchy was applied: (1) tiering exception, (2) formulary exception, (3) step therapy exception, (4) quantity limit exception, (5) payment-related issue, (6) request for prior authorization.

**eTable 2. Search terms used to classify type of coverage determination**

| **Category** | **Inclusion search terms** | **Exclusion search terms** |
| --- | --- | --- |
| Request for prior authorization | “you asked the plan to cover”, “the drug is requested to treat”, “issue a decision regarding coverage”, “at issue is coverage”, “prior authorization”, “prior auth”, “coverage criteria”, “pa requ”, “pa deni”, “authoriz”, “utilization” | N/A |
| Request for formulary exception | “formulary exception”, “not on formulary”, “non-formulary”, “non formulary”, “not formulary”, “alternative formulary”, “formulary alternatives” | “non-formulary drugs are not eligible for a tiering exception”, “unknown diagnosis” |
| Request for step therapy exception | “step therapy”, “exception to step therapy”, “step-therapy”, “try another drug before the plan” | N/A |
| Request for quantity limit exception | “quantity limit”, “quantity-limit”, “plan limits the amount”, “plan limits the quantity”, “requested quantity”, “requested amount”, “number of doses”, “requested dosage” | “quantity limit exception review is not required” |
| Request for tiering exception | “tiering exception”, “lower tier”, “same type”, “tier exception”, “tier req”, “tier appeal”, “cost-share red” | “exception to the plan's coverage criteria” |
| Request for reimbursement | “deductible”, “initial coverage stage”, “initial coverage phase”, “true out-of-pocket”, “troop”, “benefit phase”, “copayment amount”, “coinsurance amount”, “you paid”, “charged an amount” | N/A |

The column “Inclusion search terms” lists phrases that, when present, triggered a positive classification. The column “Exclusion search terms” lists phrases that, when present, overrode an otherwise positive classification (i.e., the record was not assigned that category if any exclusion term was found).

**eMethods 2. Approach to classifying reasons for unfavorable outcomes of appeals**

When appeals had unfavorable outcomes, we determined the primary reason for the appeal outcome. However, we did not systematically assess reasons for favorable outcomes, because favorable outcomes usually resulted simply from criteria for coverage being satisfied. Reasons for unfavorable outcomes of appeals included:

- Part D exclusion for weight loss: Medicare Part D is statutorily prohibited from covering drugs prescribed only for weight loss.
- Off-label use: Some appeals were denied when GLP-1s were prescribed for off-label indications.
- Utilization management requirements: Some appeals were denied because the criteria for prior authorization, formulary exceptions, step therapy exceptions, quantity limit exceptions, and tiering exceptions were not met.
- Administrative issues: A small number of appeals were denied for various miscellaneous reasons, such as a lack of information provided to the plan or Part D rules around reimbursement.

The list of search terms for classifying reasons for unfavorable outcomes was developed using a similar approach as described in eMethods 1.

When >1 reason indicator was positive, the following hierarchy was applied to determine the *primary* reason for an unfavorable appeal outcome: (1) Part D exclusion for weight loss, (2) off-label use, (3) utilization management criteria not met, (4) administrative issue.

**eTable 3. Search terms used to classify reason for unfavorable appeal outcome**

| **Category** | **Inclusion search terms** | **Exclusion search terms** |
| --- | --- | --- |
| Due to Part D exclusion for weight loss | “exclud”, “weight loss”, “weight gain”, “anorexia”, “obes” | “not being prescribed to promote weight loss”, “not being used for weight loss”, “not prescribed for weight loss” |
| Due to off-label use | “off-label”, “off label”, “non-fda”, “non fda”, “not fda”, “not a medically accepted indication”, “not being prescribed for a medically accepted”, “no citations”, “does not meet the definition of a medicare part d drug”, “medicare approved compendia do not” | N/A |
| Due to not meeting utilization management criteria | “prior authorization”, “submitted documentation does not”, “prior auth”, “prior approval criteria are not met”, “step therapy criteria are not met”, “step therapy exception criteria are not met”, “neither step therapy”, “quantity limit exception criteria are not met”, “quantity limit criteria are not met”, “dose restriction”, “plan limits the amount”, “limits the amount of”, “coverage criteria are not met”, “exception to the plan's coverage criteria is not supported”, “formulary exception criteria are not met”, “exception to the formulary”, “tiering exception criteria are not met”, “not eligible for a tiering exception”, “no available lower tier brand”, “no available lower tier biologic”, “lower cost-sharing tiers only contain generic”, “must try”, “required step therapy”, “required to try”, “has not indicated that the enrollee has tried”, “does not state why all available lower tier alternatives”, “documentation does not support why the formulary alternatives”, “alternative formulary drugs must have been”, “documentation does not show”, “formulary alternatives must”, “alternative formulary”, “alternative safer”, “requested drug at the requested amount is not eligible”, “formulary exception requirements have not been met”, “prescriber has not provided patient-specific clinical documentation”, “documentation does not”, “to grant an exception for”, “lower cost sharing tier”, “lower tier”, “an exception to step therapy criteria is not met”, “without a diagnosis”, “insufficient documentation” | N/A |
| Due to administrative issue | “not receive”, “redetermination”, “administrative”, “procedural”, “reimbursement”, “deductible”, “coinsurance”, “copay”, “true out-of-pocket”, “troop”, “GoodRx”, “manufacturer assistance”, “patient assistance”, “benefit phase”, “claim straddled”, “enhanced benefit”, “coverage stage” | N/A |

The column “Inclusion search terms” lists phrases that, when present, triggered a positive classification. The column “Exclusion search terms” lists phrases that, when present, overrode an otherwise positive classification (i.e., the record was not assigned that category if any exclusion term was found). Of note, some appeal denials explicitly mentioned Part D’s statutory exclusion of drugs used for weight loss; such denials were classified as occurring due to Part D’s exclusion for drugs used for weight loss. In contrast, some prescriptions written for an indication of obesity or overweight were denied due to off-label use.

**eMethods 3. Validation of approach to classifying types of initial coverage determinations and reasons for unfavorable outcomes**

To validate our classification of types of initial coverage determinations and reasons for unfavorable outcomes, two investigators manually classified the same random sample of 150 appeals (50 appeals per drug across three drugs). Investigators came to consensus on classifications for each appeal, and this consensus classification was then compared against the automated classifications. Agreement was high for both determination type (98.0% observed agreement; Cohen’s κ=0.96) and reason for unfavorable outcome (92.0%; Cohen’s κ=0.87).

**eTable 4. Indications for GLP-1 prescriptions among a subsample of appeals denied due to off-label prescribing in 2024 and 2025**

| **Indication** | **Injectable semaglutide (n=200)** | | **Oral semaglutide (n=177)** | | **Tirzepatide (n=200)** | |
| --- | --- | --- | --- | --- | --- | --- |
|  | **n** | **%** | **n** | **%** | **n** | **%** |
| Obesity or overweight | 129 | 65% | 100 | 57% | 129 | 65% |
| *Obesity or overweight as a sole indication* | 50 | 25% | 49 | 28% | 44 | 22% |
| Prediabetes or insulin resistance | 112 | 56% | 103 | 58% | 116 | 58% |
| Cardiovascular (including hypertension and hyperlipidemia) | 31 | 16% | 25 | 14% | 37 | 19% |
| Diabetes | 9 | 5% | 4 | 2% | 10 | 5% |
| Obstructive sleep apnea | 3 | 2% | 3 | 2% | 17 | 9% |
| Polycystic ovarian syndrome | 1 | 1% | 1 | 1% | 1 | 1% |
| Liver disease | 3 | 2% | 1 | 1% | 3 | 2% |
| Other specified condition | 8 | 4% | 2 | 1% | 5 | 3% |
| No condition specified | 4 | 2% | 2 | 1% | 1 | 1% |

We manually classified indications for a subsample of GLP-1 prescriptions into the categories in this table. This table reports the indications for appeals denied due to off-label prescribing in 2024 and 2025. We provide this information for a random subsample of such appeals: 200 for injectable semaglutide, 177 for oral semaglutide (there were only 177 denied appeals due to off-label prescribing during this time period for this drug), and 200 for tirzepatide. Prescriptions could have multiple indications listed, so *indications in this table are not mutually exclusive*. However, this table does include the count of appeals denied due to off-label prescribing for which obesity/overweight was the sole indication listed (“Obesity or overweight as a sole indication”). The “diabetes” category included appeals where the prescription indication was listed as diabetes (unspecified type), type 1 diabetes, or other types (e.g., gestational diabetes or post-transplant diabetes).
